# Effect of high nitrate vegetable juice supplementation on plasma nitrate and blood pressure in adults: a pilot randomized crossover intervention in healthy volunteers

**DOI:** 10.1017/jns.2022.34

**Published:** 2022-06-06

**Authors:** Nour A. Elsahoryi, Christopher Cardwell, Sarah Gilchrist, Jayne V. Woodside

**Affiliations:** 1 Centre for Public Health, School of Medicine, Dentistry and Biomedical Science, Queen's University Belfast, Belfast, UK; 2 Department of Nutrition, Faculty of Pharmacy & Medical Sciences, University of Petra, Amman, Jordan

**Keywords:** Beetroot, Hypertension, Nitrate, Vegetable juice, BP, blood pressure, SBP, systolic blood pressure, DBP, diastolic blood pressure, BRJ, beetroot juice, GLVJ, green leafy vegetable juice

## Abstract

Beetroot juice (BRJ) has been demonstrated to decrease blood pressure (BP) due to the high inorganic nitrate content. This pilot randomized crossover trial aimed to investigate the effect of two different high nitrate vegetable juices on plasma nitrate concentrations and BP in healthy adults. Eighteen healthy volunteers were randomized to receive 115 ml of BRJ or 250 ml of green leafy vegetable juice for 7 d which contained similar amounts of nitrate (340 mg) daily. Blood samples were collected, and clinic BP measured at baseline and at the end of each juice consumption. Daily home BP assessment was conducted 2 h after juice consumption. Nitrate and nitrite concentrations were analysed using a commercially available kit on a Triturus automated ELISA analyser. Hills and Armitage analysis was used for the two-period crossover design and paired sample *t*-tests were performed to compare within-group changes. Plasma nitrate and nitrite concentrations significantly increased and there was a significant reduction in clinic and home systolic blood pressure (SBP) mean during the BRJ period (*P*-values 0⋅004 and 0⋅002, respectively). Home diastolic blood pressure (DBP) reduced significantly during green leafy vegetable juice consumption week (*P*-value 0⋅03). The difference between groups did not reach statistical significance during the formal crossover analysis adjusted for period effects. BRJ and green leafy vegetable juice may reduce SBP or DBP, but there was no statistically significant difference between the two juices, although this was only a pilot study.

## Graphical abstract

**Figure:**
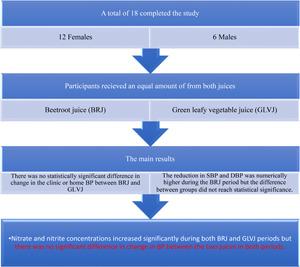

## Background

Vegetables represent the main dietary source of nitrate and contribute 85 % of daily nitrate intake, while other sources of nitrate come from other foods, such as meat and water^(1,2)^. The amount of nitrate varies between vegetables and also depends on many factors, such as soil type, genotype, growth condition, storage and transport conditions^(1,2)^. For example, celery, cress, chervil, lettuce, red beetroot, spinach and rocket contain more than 250 mg/100 g of fresh weight, while broccoli, carrot, cauliflower, cucumber, pumpkin and chicory contain less than 20 mg/100 g of fresh weight^(1)^. Many studies have suggested that inorganic nitrate can have health benefits on cardiovascular disease (CVD) in general and hypertension in particular via the nitrite–nitric oxide (NO) pathway^(3–6)^. As beetroot is a rich source of dietary inorganic nitrate^(7)^, a large number of intervention studies have demonstrated a positive effect of beetroot juice (BRJ) on blood pressure (BP) and, therefore, CVD risk^(8–13)^.

Most studies have focused on the acute effect of BRJ on BP^(9,13–19)^, while few studies have examined the effect of chronic nitrate consumption on BP^(20–22)^. The first study that tested the chronic effects of sodium nitrate on BP was a crossover study in healthy volunteers with 3 d supplementation and 10 d washout^(22)^. The results reported a significant reduction in diastolic blood pressure (DBP) by −3⋅7 mmHg^(22)^. Since then the interest in the effects of dietary nitrate on cardiovascular health has grown, and several studies have confirmed the lowering effects of dietary nitrate on chronic and acute BP, which have been reviewed^(2)^. A systematic review and meta-analysis of 16 trials (both acute and chronic in design) suggested that high inorganic nitrate supplementation (inorganic nitrate supplementation and via increased beetroot or BRJ intake) reduced systolic blood pressure (SBP) by −4⋅4 mmHg, while DBP was not affected, and the findings in this meta-analysis were homogeneous^(7)^. A more recent systematic review and meta-analysis of nine crossover trials and three parallel trials also reported that inorganic nitrate and beetroot consumption produced beneficial effects on endothelial function^(23)^. Another systematic study and meta-analysis of 34 studies found that, in the acute setting, inorganic nitrate consumption decreased SBP by −4⋅8 mmHg and DBP by −1⋅74 mmHg, as well as improving endothelial function, reducing atrial stiffness, and reducing platelet aggregation^(24)^. Recently, Bonilla Ocampo *et al.* reported that BRJ intervention could significantly decrease the risk of suffering cardiovascular events and that it should be promoted as a key component of a healthy lifestyle to control BP in healthy and hypertensive individuals^(17)^. In contrast, the most recent systematic review and meta-analysis of twelve randomized control trials examining the effect of BRJ on BP concluded that there was no change either on SBP or DBP in the intervention groups compared with the control group^(19)^; therefore, there is still some inconsistency in the literature.

Although there are several other nitrate-rich food sources, including green leafy and root vegetables^(25)^, research on the pharmacokinetic and physiological effects of nitrate supplementation has mainly used either sodium nitrate or BRJ as a nitrate donor. Very few studies have investigated the effect of more than one high nitrate source^(10,26)^. A recent crossover study found that (beetroot, rocket salad or spinach) juice increased plasma nitrate and lowered BP to a greater extent than sodium nitrate^(26)^ after a single exposure. A further crossover trial investigated the effect of a high nitrate diet and control diet on BP for 1 week^(3)^. The high nitrate diet consisted of the consumption of five different types of green leafy vegetables (salad, such as lettuce, rocket, celery, leeks, fennel and mixed salad leaves), while on the control arm, participants had to avoid these for 7 d. The results for BP indicated that consuming two portions of a variety of high nitrate vegetables daily over 7 d reduced SBP (by 4 mmHg) in healthy, normotensive young women, but had no significant effect on DBP^(3)^.

This study aimed to examine the feasibility of carrying out a randomised crossover intervention study of two different high nitrate vegetable juices, consumed over 1 week, on plasma nitrate levels and BP in healthy adults. In this study, we hypothesised that the oral supplementation of BRJ and green leafy vegetable juice (GLVJ) with similar amounts of nitrate would decrease BP in healthy participants and that the effect on BP would be independent of the BP measurement protocol (clinic measurement and home measurement).

## Materials and methods

### Participants

A total of 18 non-smoking subjects between 30 and 65 years of age were recruited between November 2016 and March 2017. Participants were excluded if they had medical conditions (i.e., diabetes, acute coronary syndrome or transient ischaemic attack within the past 3 months, using oral anticoagulation therapy), if they reported excessive alcohol consumption (>14 U/week), if they had BMI of >35 kg/m^2^, and if they were pregnant or lactating or had dietary restrictions (such as special diets or such as a low nitrate diet) that could interfere with the nutritional intervention and study outcomes. Written informed consent was obtained from all participants before participation in the study. The study was approved by the School of Medicine, Dentistry and Biomedical Sciences Research Ethics Committee of Queen's University Belfast and adhered to the guidelines contained within the Declaration of Helsinki. As this was a pilot study, formal sample size calculations were not performed.

### Study design

**Fig. 1.**
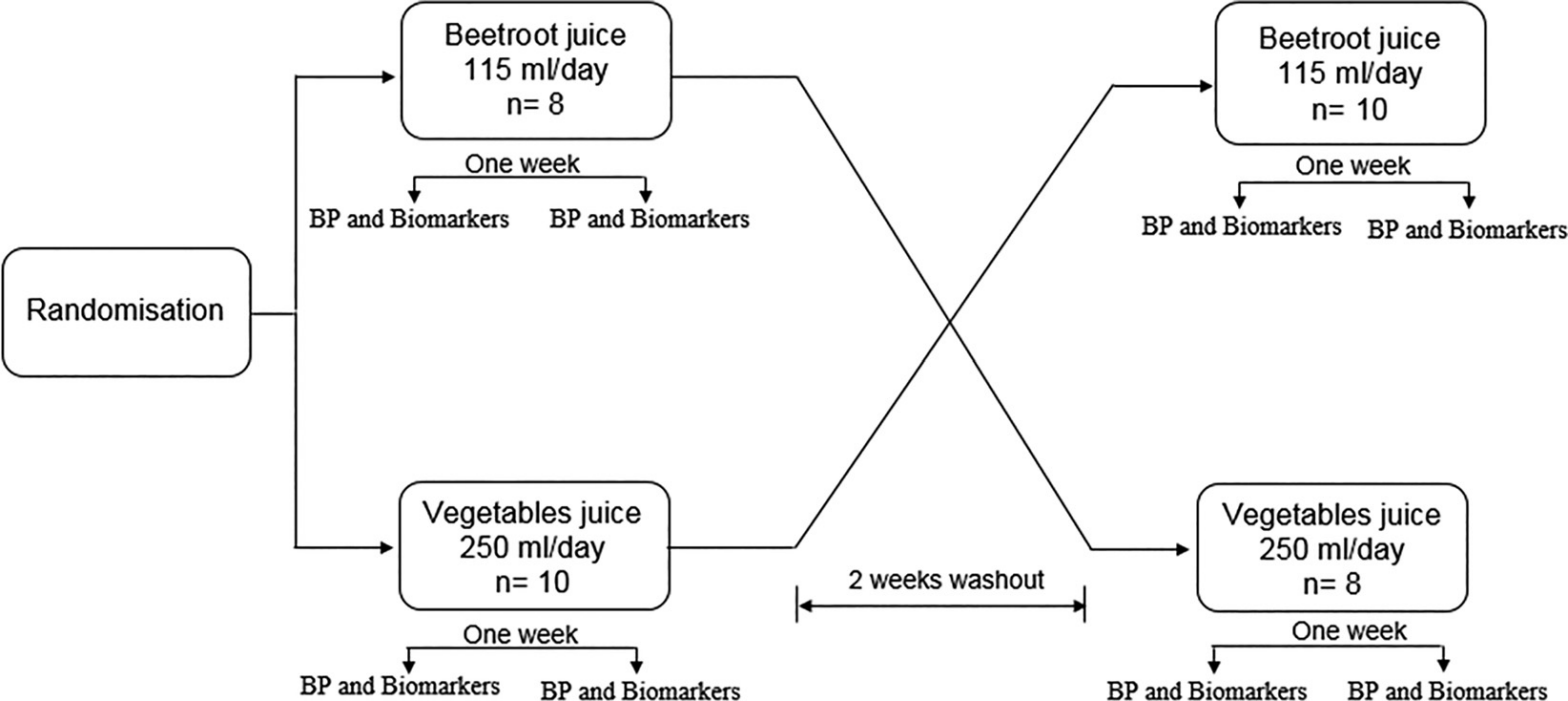
Overview of study design.

The study was a 1-week, randomized crossover trial (Fig. 1). Participants were randomized either to BRJ or GLVJ by use of a block design (block size *n* 4; www.randomization.com) for 1 week, with a 2-week washout between the two interventions.

The registration number is NCT04736784. The first trial registration date on the website is 03 February 2021. The actual study start date is 30 January 2017, and the actual study completion date is 15 May 2017. The source of the registration is *ClinicalTrials.gov*.

### Study interventions

The intervention was designed to give the participants an equal amount of nitrate (approximately 340 mg of nitrate daily) from both juices. Therefore, participants were randomized to receive 115 ml of BRJ or 250 ml of GLVJ daily for 7 d. Both juices were made in the UK. The juices had to be kept refrigerated at 0–5°C and once opened it had to be consumed within 48 h. The research team provided the participants with the appropriate amounts of juice for the duration of the intervention. All packages were stored in suitable boxes for refrigerated storage.

The amount of nitrate in the BRJ and in the leafy juice was estimated based on previous research^(1,27,28)^. Furthermore, the nutritional compositions of the BRJ^(29,30)^ and the GLVJ^(31)^ were reported in previous studies. The label suggested the nutritional content of the BRJ included 34 kcal, 0 g fat, 7⋅6 g carbohydrates, 0⋅8 g protein and 0⋅08 g salt/100 ml. The nutritional content of the GLVJ included 13 kcal, 0⋅2 g fat, 2 g carbohydrates, 0⋅5 g protein and 8 mg sodium/100 ml.

The leafy vegetable juice contained 30 % celery, 30 % cucumber, 15 romaine, 15 % spinach, 5 % kale, 2⋅5 % lemon and 2⋅5 ginger. The calculated amount of nitrate in the GLVJ (340⋅30 mg/250 ml) was less than the amount of nitrate in the BRJ (735⋅75 mg/250 ml). Therefore, the participants were asked to drink 250 ml of the GLVJ daily for 7 d and 115 ml of the BRJ daily for 7 d. Previous studies have suggested that the peak action time for dietary nitrate to reduce BP is ~2⋅5–3 h after ingestion^(32)^. Therefore, participants were requested to drink both juices in the evening, 2–3 h before measuring home BP.

Participants were advised to follow their usual diet and not to make any changes to dietary or lifestyle habits and instructed that their body weight was to remain stable for the duration of the intervention. They were also advised to avoid mouthwash during the study, as an antibacterial mouthwash has been shown to attenuate the rise in plasma nitrite^(3)^.

### Outcomes

Height was assessed at baseline using a Leicester portable height measure. Before and after each intervention period, participants were weighed using recently calibrated digital weighing scales (Seca 876, SECA, Birmingham, UK), and BP was measured.

Questionnaires to assess physical activity and the education level were completed at baseline. General physical activity was assessed (i.e. over the last 4 weeks) using the general practice physical activity questionnaire (GPPAQ). The primary endpoint of this study was to determine the effect of two different high nitrate juices on the clinic- and home-measured BP.

## Blood pressure measurements

### Clinic BP measurements

With the patient seated comfortably for 15 min before measurement and the arm supported at the level of the heart, clinical BP was measured in triplicate by using an automated BP monitor (Omron Healthcare, The Netherlands). The final value was calculated by the average from the second and third readings.

### Daily home BP monitoring

An automated BP monitor (Omron Healthcare, The Netherlands) was provided to each participant to measure daily resting BP at home. Participants were asked to conduct duplicate measurements, in a seated position, at the evening, 2 h after juice consumption and before going to bed. Participants were trained on how to use the monitor, with an emphasis on the correct positioning of the monitor and arm. In addition, written instructions on measuring BP were provided. A form was provided to participants to record the BP readings (SBP and DBP) and the time of each measurement. The average of the measurements was calculated; the average of the values for each week was calculated to evaluate differences in BP between the two interventions. To increase participant compliance, text messages were sent to the participants, as a reminder, daily, during the study before juice drinking to drink the juice and after juice drinking to record BP values and other messages as a reminder before each lab visit.

### Blood sample collection

A total of 32 ml of non-fasting whole blood (collected in the morning) collected into serum and EDTA tubes were obtained from participants using standard phlebotomy procedures before and after each intervention period. All participants were seated for venepuncture. Samples were centrifuged at 3000 rpm for 15 min at 4°C and separated appropriately for the proposed assays. Sample aliquots were stored at −80°C until analysis.

### Analysis of plasma nitrate and nitrite concentrations

Plasma nitrate concentrations were quantified using a commercial kit and calibrators from R&D Systems (Minneapolis, MN, USA) according to the manufacturer's instructions. Kits were run on a Triturus automated ELISA analyser supplied by Grifols UK Ltd. Plasma samples were filtered using Amicon Ultra 2, 10 000 MW centrifugal filter units (Millipore UK Ltd). The assay procedure measured total nitrite by converting nitrate to nitrite. Samples and standards, as well as quality controls, were prepared as per the manufacturer's instructions. The plate was then incubated at room temperature for 10 min, and samples were read at 540 nm with wavelength correction at 690 nm.

### Participant's view of the intervention juices

A series of 5-point Likert scales were used to assess ease of consumption of the intervention juices in the following question: based on your experience in this study, did you find the juice (either BRJ or GLVJ based on the intervention week): very easy, easy, sometimes hard sometimes easy, hard, and very hard. The same question was asked to assess the acceptability with the following response choices: very acceptable, acceptable, unsure, unacceptable, and very unacceptable. In addition, participants were asked if drinking the juice for 7 d is shown to help improve BP: (1) I would be willing to continue drinking the juice after the study finished and (2) I would be willing to spend approximately £2⋅25 pound/week for the BRJ or £14⋅00/week for the GLVJ and how much they agreed with the statements given, with the following responses: strongly agree, agree, unsure, disagree, and strongly disagree.

In addition, open and closed questions were also used such as whether the participants found the study products gave any side effects and if they would be willing to continue taking the products post-intervention if found to be beneficial to health. For example, ‘Did you experience any side effects when drinking (juice name), during the study’, ‘If you found drinking (juice name) over a period of seven days hard or unacceptable, please tell us why’. Two separate forms were used, one for each juice after each intervention week.

### Statistical analysis

The data were checked for normality by using the Kolmogorov–Smirnov test. Descriptive statistics were obtained for all variables. Normal variables were reported as mean (sd) and categorical variables were reported as percentages. Descriptive statistics and paired *t*-test was performed using SPSS v17⋅0 for Windows (SPSS Inc., Chicago, IL, USA). Paired sample *t*-tests were performed on BP data to compare the within-group effect of each juice before and after each intervention. Hills and Armitage analysis^(33)^ was used for a two-period crossover design using STATA software (version 9.0, StataCorp, TX, USA) and Excel (Windows 2013). Treatment effects (difference in mean adjusted for the period) and 95 % CI were calculated. Values of *P* < 0⋅05 (2-tailed) were considered statistically significant.

## Results

### Recruitment and baseline

A total of eighteen (twelve females and six males) were completed the study. Table 1 shows the baseline characteristics for all study participants and separately according to the assigned intervention group. Based on the randomisation, eight participants started with the BRJ and ten participants started with the GLVJ. The mean age of the study participants was 40⋅9 years. The baseline physical activity category for most participants was moderate physical activity level. The education level was postgraduate/high degree level for 61 % of the participants.

**Table 1.** Baseline characteristics of participants randomized to the BRJ and the GLVJ according to the assigned intervention group

First juice	BRJ first (*n* 8)	GLVJ first (*n* 10)
Age	45⋅25 ± 12⋅71	37⋅40 ± 4⋅55
Male (%)	1 (5⋅6 %)	10 (55⋅6 %)
Female (%)	17 (94⋅4)	8 (44⋅4 %)
BMI baseline BRJ (kg/m^2^)	26⋅45 ± 4⋅89	24⋅84 ± 3⋅58
BMI baseline GLVJ (kg/m^2^)	26⋅68 ± 4⋅86	24⋅82 ± 3⋅61
Lab SBP baseline BRJ (mmHg)	116⋅13 ± 10⋅18	122⋅30 ± 11⋅36
Lab DBP baseline BRJ (mmHg)	69⋅88 ± 6⋅01	76⋅35 ± 8⋅61
Lab SBP baseline GLVJ (mmHg)	111⋅44 ± 10⋅04	123⋅85 ± 13⋅51
Lab DBP baseline GLVJ (mmHg)	64⋅81 ± 6⋅43	80⋅45 ± 6⋅47
Home SBP baseline BRJ (mmHg)	109⋅02 ± 13⋅62	119⋅88 ± 10⋅5
Home DBP baseline BRJ (mmHg)	65⋅72 ± 6⋅02	73⋅27 ± 6⋅95
Home SBP baseline GLVJ (mmHg)	105⋅68 ± 12⋅02	118⋅39 ± 10⋅33
Home DBP baseline GLVJ (mmHg)	67⋅71 ± 6⋅16	70⋅92 ± 6⋅87
Physical level		
Active	1 (12⋅5 %)	4 (40 %)
Moderate	5 (62 %)	2 (20 %)
Inactive	2 (25 %)	4 (40 %)
Education level		
GCSE*	1 (12⋅5 %)	1 (12⋅5 %)
A-level	1 (12⋅5 %)	1 (12⋅5 %)
Diploma/certificate	1 (12⋅5 %)	2 (20 %)
Postgraduate/high degree	5 (62⋅5 %)	6 (60 %)

Normal distribution is presented as mean (sd) for normal distributed variables and (*n*%) for categorical variable.

BMI, body mass index; BP, blood pressure; SBP, systolic blood pressure; DBP, diastolic blood pressure; BRJ, beetroot juice; GLVJ, green leafy vegetable juice.

* GCSE/O-Level/intermediate/junior/group certificate.

### Effect of BRJ *v.* GLVJ intervention on SBP, DBP and serum nitrate and nitrite concentrations

As shown in Table 2, there was no statistically significant difference in change in the clinic or home BP between BRJ and GLVJ during the formal crossover analysis adjusted for period effects. The reduction in SBP and DBP was numerically higher during the BRJ period, but the difference between groups did not reach statistical significance. The results of the Hills and Armitage analysis also indicated that there was an increase in nitrate and nitrite concentrations during both interventions, but, again, there were no statistically significant differences between interventions. The mean difference in BMI, BP (clinic and home) and nitrate during each intervention period is shown in Table 3. The results indicated that there was no significant change in BMI during each intervention. For BP, the result indicated that there were statistically significant reductions in the clinic and home SBP mean during the BRJ period (*P*-values 0⋅004 and 0⋅002, respectively), while the reduction in home DBP was statistically significant during the GLVJ intervention (*P*-value 0⋅03). Nitrate and nitrite concentrations increased significantly during both BRJ and GLVJ periods.

**Table 2.** The effect of BRJ and GLVJ on the clinic BP and nitrate for the healthy adults

BP	BRJ	GLVJ	Treatment effect*	95 % CI	*P*-value
	Mean change (sd)	Mean change (sd)	Mean difference in change		
Clinic SBP (mmHg)	−6.06 ± 7.76	−1.89 ± 12.60	−4.83	−11.56, 1.90	0.15
Clinic DBP (mmHg)	−2.14 ± 8.42	0.92 ± 7.83	−3.62	−8.56, 1.33	0.14
Home SBP (mmHg)	−3.93 ± 12.94	−8.41 ± 14.57	−2.76	−9.15, 3.65	0.38
Home DBP (mmHg)	−1.40 ± 5.29	3.25 ± 5.18	3.27	−0.25, 6.80	0.07
Nitrate (μmol/l)	20.18 ± 9.95	17.34 ± 3.65	−7.24	−19.91, 5.44	0.24
Nitrite (μmol/l)	4.09 ± 6.07	0.65 ± 19.07	−6.75	−19.49, 5.99	0.28

BP, blood pressure; SBP, systolic blood pressure; DBP, diastolic blood pressure; BRJ, beetroot juice; GLVJ, green leafy vegetable juice.

* Treatment effect was adjusted for the period.

**Table 3.** The mean change in BMI, BP (clinic and home), nitrate and nitrite concentrations for participants randomised for BRJ and GLVJ

	Pre-BRJ	Post-BRJ		Pre-GLVJ	Post-GLVJ	
	Mean (sd)	Mean (sd)	*P*-value	Mean (sd)	Mean (sd)	*P*-value
BMI (kg/m^2^)	25⋅56 ± 0⋅98	25⋅62 ± 0⋅96	0⋅32	25⋅65 ± 0⋅99	25⋅64 ± 0⋅97	0⋅88
Clinic SBP	119⋅56 ± 11⋅00	113⋅50 ± 9⋅54	0⋅004	118⋅33 ± 13⋅36	118⋅69 ± 11⋅64	0⋅92
Clinic DBP	73⋅47 ± 8⋅06	71⋅33 ± 6⋅26	0⋅30	73⋅50 ± 10⋅15	74⋅42 ± 6⋅61	0⋅63
Home SBP	115⋅05 ± 12⋅87	110⋅47 ± 13⋅08	0⋅002	112⋅74 ± 12⋅58	112⋅43 ± 11⋅23	0⋅90
Home DBP	69⋅91 ± 7⋅45	69⋅08 ± 8⋅12	0⋅49	69⋅49 ± 6⋅58	66⋅89 ± 7⋅13	0⋅03
Nitrate	13⋅78 ± 4⋅80	35⋅68 ± 23⋅18	≤0⋅001	12⋅11 ± 3⋅25	30⋅46 ± 12⋅25	≤0⋅001
Nitrite	14⋅06 ± 4⋅79	35⋅83 ± 23⋅14	≤0⋅001	12⋅41 ± 3⋅29	30⋅74 ± 12⋅25	≤0⋅001

Statistical significance was determined by the paired sample *t*-test. BP unit is mmHg. Nitrate and nitrite unit is μmol/l.

BP, blood pressure; SBP, systolic blood pressure; DBP, diastolic blood pressure; BRJ, beetroot juice; GLVJ, green leafy vegetable juice.

### Acceptability

At the end of each intervention week, acceptability and willingness were assessed. The results indicated that most participants, who suffered from the side effect, reported urine colour changes after drinking BRJ and bowel habit changes after drinking GLVJ, as shown in Table 4. However, none of the side effects mentioned were serious and none of the participants needed to seek medical advice or discontinue participation in the study. The majority of participants rated both juices as easy to consume. Participants reported that GLVJ was unacceptable (5⋅6 %) and 16⋅7 % of the participants reported that GLVJ was very unacceptable due to the taste and odd smell and due to changes in bowel habit. In both interventions, 33⋅3 % (same percentage in both interventions) of the participants were willing to consume/purchase the study products post-intervention if the products could be demonstrated to improve heart health.

**Table 4.** Responses to the tolerability of study products and willingness to consume/purchase post-intervention according to the juice type

		BRJ (%)	GLVJ (%)
Side effects	Yes	72⋅2 %	16⋅7 %
Side effects reported	Change in:	Urine colour, bloating	Bowel habit
	No	27⋅8 %	83⋅3 %
Ease of consumption	Very easy	11⋅1 %	0⋅0 %
	Easy	50⋅0 %	50⋅0 %
	Sometimes hard/sometimes easy	16⋅7 %	27⋅8 %
	Hard	16⋅7 %	11⋅1 %
	Very hard	5⋅6 %	11⋅1 %
Acceptability	Very acceptable	5⋅6 %	0⋅0 %
	Acceptable	72⋅2 %	72⋅2 %
	Unsure	5⋅6 %	5⋅6 %
	Unacceptable	11⋅1 %	5⋅6 %
	Very unacceptable	5⋅6 %	16⋅7 %
Willingness to continue post-intervention	Strongly agree	11⋅1 %	16⋅7 %
	Agree	33⋅3 %	33⋅3 %
	Unsure	16⋅7 %	22⋅2 %
	Disagree	22⋅2 %	16⋅7 %
	Strongly disagree	16⋅7 %	16⋅7 %
Willingness to purchase	Strongly agree	16⋅7 %	5⋅6 %
	Agree	33⋅3 %	33⋅3 %
	Unsure	16⋅7 %	5⋅6 %
	Disagree	11⋅1 %	16⋅7 %
	Strongly disagree	22⋅2 %	27⋅8 %

BRJ, beetroot juice; GLVJ, green leafy vegetable juice.

## Discussion

This is the first study that has determined the effect of two different high nitrate juices on chronic BP, measured both as home BP and clinic BP. Our results indicated that, after BRJ consumption, home and clinic SBP reduced significantly while, after GLVJ consumption, home DBP reduced significantly. The crossover analysis, however, indicated that there was no statistically significant difference between the BRJ and GLVJ interventions, although, as this was a pilot study, we were probably not powered to detect this.

These findings are consistent with some crossover studies^(3,10)^ that compared home and clinic BP, and our findings are close to those of a randomized study that compared three different BP measurement methods (clinic BP, daily home BP monitoring, and 24-h ambulatory blood pressure monitoring (ABPM))^(10)^. The authors looked for the effect of BRJ and blackcurrant juice on BP for twenty-four non-smoking subjects. The control group drink 200 ml of blackcurrant juice containing 2⋅7 ± 0⋅1 mg of nitrate per bottle. The results reported that BP measured by all methods reduced during the intervention, but that the changes in clinic BP, 24-h ABPM and daily home BP monitoring were not statistically significant between the two interventions^(10)^. Moreover, the results of this study agree with very recent intervention studies^(15,16)^. Researchers examined nitrate supplementation on microvascular and large-vessel EF and BP in a randomized, double-blind, placebo-controlled pilot study in healthy older adults and they concluded that BRJ (a 70 ml NO_3_-rich BRJ drink) ingestion for 28 (±7) d potentially improves BP and large-vessel EF in healthy older adults^(15)^. Acute BR supplementation increased plasma nitrite concentrations and reduced SBP and DBP in both older and younger adults in a double-blind, crossover study, and the study groups consumed either 150 ml of nitrate-rich BRJ (BR; 10⋅5 mmol nitrate) or placebo (PL; 1 mmol nitrate)^(16)^.

Using clinic BP is the most commonly used method, but its poor reliability is universally recognised, due to measurement bias associated with white coat syndrome, standardisation of protocol and operator bias^(34)^. Home BP readings may provide more reproducible results due to a lack of white coat syndrome,^(35)^ but, on the other hand, home BP monitors do not provide nocturnal recordings and therefore cannot give information on diurnal patterns in BP, which are more prevalent to the cardiovascular risk factors and the accuracy of devices remains a limiting factor associated with home BP monitors^(36)^. Therefore, measuring BP on a continuous basis (ambulatory BP monitoring) can detect abnormal fluctuations in BP that might go unnoticed when BP is only measured in the doctor's office. Our trial examined the effect of two high nitrate juices on BP in healthy individuals in the absence of dietary restrictions, such as a low nitrate diet. Dietary restrictions in studies with high nitrate juice and BP remove confounding dietary factors that may have an effect (negative or positive) on BP, thus making the interpretation of study results more straightforward. The drawback, however, is that BRJ and GLVJ as nutritional interventions to regulate BP would likely be consumed as part of a normal diet, not as part of a low nitrate diet, or in the absence of other dietary components (i.e. coffee, alcohol and black tea) that may affect BP^(13)^. It would, therefore, be uncertain whether there is any clinically relevant benefit from BRJ or GLVJ on BP in the unregulated home environment. Monitoring of background diet would have allowed us to comment more definitively on any other concurrent dietary changes that may have affected our outcomes, and this must be considered a limitation of the study.

Comparing the results after and before each intervention, the results of BRJ agreed with many previous studies that support the hypothesis that a high nitrate diet, mainly as BRJ, can reduce BP. A recent systematic review and meta-analysis (2018) of eleven randomized control trials reported that BRJ has a positive effect in reducing BP compared with the control group mainly on SBP^(17)^. An older meta-analysis of sixteen trials showed that inorganic nitrate and BRJ supplementation were associated with a significant reduction in SBP, whereas no significant effect was observed in DBP^(7)^. In addition, a review of human intervention studies involved studies in the chronic and acute setting and reported that there was an inverse relationship between the dose of nitrate consumed and the corresponding reduction in SBP (by 3 mmHg with doses of nitrate as low as 3 mmol of nitrate) in addition to the beneficial effects of dietary nitrate on endothelial function^(23)^. However, in contrast, Remington and Winters reported that there was no effect of BRJ on BP in twelve randomized control trials^(19)^. For GLVJ, to our knowledge, this is the first study to determine the effect of such a juice on BP in a chronic setting. The juices given should have had similar nitrate content but may have differed in levels of other nutrients, such as betaine and polyphenols, which could impact BP-lowering potential. The findings show some effects of GLVJ on BP (home DBP), but a potential lack of power to detect differences between the BRJ and GLVJ juice groups means it is difficult to draw conclusions from this pilot study. Therefore, more studies are required in this area, and data from this study can be used to power these appropriately. Finally, in response to the levels of liking questionnaire, participants generally rated their liking of both juices acceptable and easy to consume. Both interventions were well tolerated. In addition, most participants were willing to consume them and purchase them if studies demonstrate the beneficial effects of either juice on cardiovascular health including BP. The most commonly self-reported adverse effect with BRJ was red urine (beeturia) and this agreed with other beetroot studies^(10,37)^. Some volunteers experienced mild, temporary abdominal discomfort from GLVJ; however, no serious adverse events were reported.

## Strengths and limitations

There were several strengths in this pilot study: firstly, general strength points where it was a crossover randomised intervention; BRJ and GLVJ were supplied to participants to ensure limited costs were incurred by the participants, and advice and support were given at the beginning and throughout the study to ensure participants understood fully the requirements for involvement. Secondly, text messages were used to encourage good participant compliance and to monitor compliance and minimise dropouts over the course of the study: for example, daily messages were sent before juice drinking to drink the juice and after juice drinking to record BP values, and other messages were sent as a reminder before each lab visit. Finally, participants had no restrictions on their usual diet; therefore, requirements for the study were not intensive, so it would have been easy for participants to stick to their usual routine and not have too many demands imposed by the study protocol.

On the other hand, this study also had limitations: first, the small sample size and short duration of this trial may have reduced the power of the study to detect significant differences in change in BP between the intervention groups, although it was designed as a pilot study. Secondly, participants may have forgotten to drink the juice or drunk less than the appropriate amount, despite efforts to maximise compliance. Third, when deciding on how much juice each participant should drink, we used nitrate content from the literature which shows marked variation and did not measure the nitrate content of the juices directly. The nitrate level in foods depends on different factors, including growth conditions, processing and food storage, making the calculations of the nitrate levels based on the literature prone to under/overestimations. Therefore, nitrate concentration estimates were potentially unreliable. Fourth, the juice volume was not the same, which may have affected the fluid volume in the body or the concentration of some nutrients. Fifth, the randomisation was not stratified for gender, and some trials of beetroot and BP reported a relationship between gender and the effect of beetroot on BP^(9,13,20)^. Finally, by comparing two nitrate-rich juices, we did not have a control juice (low in nitrate) within our study design, which would have been ideal; furthermore, as already acknowledged, we did not assess background diet to monitor any other changes that might have influenced our findings. In addition, although we did not observe any acute serious adverse effects, a few participants reported minor gastrointestinal complaints after ingestion of the juices. However, this was only a pilot study; therefore, it may be helpful to perform a retrospective power calculation to inform a larger study and conduct it for a longer time frame to allow for further investigation.

## Conclusions

In conclusion, analysis of a two-period crossover trial testing the consumption of BRJ and GLVJ by healthy volunteers for 7 d indicated that there was no significant difference in change in BP between the two juices in both periods. In addition, within-group analysis of the results indicated that clinic and home SBP reduced significantly after BRJ consumption and home DBP reduced significantly after GLVJ consumption. Based on the acceptance and tolerability assessment, this study approved the feasibility of carrying a larger, appropriately powered intervention study to investigate the effect of the used juices (BRJ and GLVJ) on BP as tolerability was overall acceptable for both juices during the intervention. Additional studies with high nitrate vegetable juice, where the studied drinks are biochemically characterized, in larger groups of healthy people are needed to determine whether they affect BP and explore the mechanism of action.
